# Construction and evaluation of a prognostic risk model of tumor metastasis-related genes in patients with non-small cell lung cancer

**DOI:** 10.1186/s12920-022-01341-6

**Published:** 2022-09-02

**Authors:** Huan Ding, Li Shi, Zhuo Chen, Yi Lu, Zhiyu Tian, Hongyu Xiao, Xiaojing Deng, Peiyi Chen, Yue Zhang

**Affiliations:** 1 Changchun University of Traditional Chinese Medicine, No. 1035 Boshuo Road, Jingyue National High-Tech Industrial Development Zone, Changchun, 130117 China; 2 Affiliated Hospital of Changchun University of Chinese Medicine, No. 1478, Gongnongda Road, Changchun, 130021 China; 3 Jilin Provincial Cancer Hospital, No. 1066, Jinhu Road, Changchun, 130021 China

**Keywords:** NSCLC, Prognosis, Metastasis-related genes, Overall survival

## Abstract

**Background:**

Lung cancer is a high-incidence cancer, and it is also the most common cause of cancer death worldwide. 80–85% of lung cancer cases can be classified as non-small cell lung cancer (NSCLC).

**Methods:**

NSCLC transcriptome data and clinical information were downloaded from the TCGA database and GEO database. Firstly, we analyzed and identified the differentially expressed genes (DEGs) between non-metastasis group and metastasis group of NSCLC in the TCGA database, Gene Ontology (GO), Kyoto Encyclopedia of Genes and Genomes (KEGG) were consulted to explore the functions of the DEGs. Thereafter, univariate Cox regression and LASSO Cox regression algorithms were applied to identify prognostic metastasis-related signature, followed by the construction of the risk score model and nomogram for predicting the survival of NSCLC patients. GSEA analyzed that differentially expressed gene-related signaling pathways in the high-risk group and the low-risk group. The survival of NSCLC patients was analyzed by the Kaplan–Meier method. ROC curve was plotted to evaluate the accuracy of the model. Finally, the GEO database was further applied to verify the metastasis‑related prognostic signature.

**Results:**

In total, 2058 DEGs were identified. GO functions and KEGG pathways analysis results showed that the DEGs mainly concentrated in epidermis development, skin development, and the pathway of Neuro active ligand -receptor interaction in cancer. A six-gene metastasis-related risk signature including C1QL2, FLNC, LUZP2, PRSS3, SPIC, and GRAMD1B was constructed to predict the overall survival of NSCLC patients. The reliability of the gene signature was verified in GSE13213. The NSCLC patients were grouped into low-risk and high-risk groups based on the median value of risk scores. And low-risk patients had lower risk scores and longer survival time. Univariate and multivariate Cox regression verified that this signature was an independent risk factor for NSCLC.

**Conclusion:**

Our study identified 6 metastasis biomarkers in the NSCLC. The biomarkers may contribute to individual risk estimation, survival prognosis.

## Introduction

Lung cancer is the leading cause of cancer-related death throughout the world. According to the World Health Organization (WHO), 2.2 million new lung cancer cases and 1.8 million fatalities are expected in 2020 [1]. NSCLC is the most common kind of lung cancer, accounting for 85 percent of all cases. Lung cancer patients die from invasiveness and metastasis in over 90% of cases, resulting in a 5-year survival rate of barely 15% [2]. As a result, research into the major regulators of metastasis is crucial for improving lung cancer treatment.

Because 80–85 percent of patients are first identified with either unresectable or metastatic tumors, despite breakthroughs in diagnostic tools, radiotherapies, and systemic treatments for NSCLC, the five-year overall survival (OS) remains at 10% [3–5]. The five-year survival rate post-operation was 20% for the tiny number of patients with a resectable and confined malignancy [3]. The prognosis of individuals with NSCLC is commonly believed to be determined by metastasis [2, 6]. Patients with metastasis have a shorter survival rate than those with localized malignancies, with just 6–8 months on average [7, 8]. The brain is the most common distant metastatic site for NSCLC [6, 9]. As a result, it's important to investigate possible biomarkers that might differentiate individuals with a poor prognosis based on tumor metastasis-related genes.

The most likely site of lung cancer metastasis is the brain, the development of the modified extracellular matrix (ECM), angiogenesis for micro-metastatic, and the building of immune escape are all part of the NSCLC metastasis process[10]. Lung cancers are exceedingly varied at both the cellular and molecular levels, according to previous research [11, 12]. Molecular markers are becoming more important in predicting the prognosis of individuals with NSCLC [12, 13]. Many prognostic models with excellent predictive value have been constructed using public resources such as The Cancer Genome Atlas (TCGA), International Cancer Genome Consortium (ICGC), and Gene Expression Omnibus (GEO) [14–16]. By mining the TCGA data, Dong et al. recently demonstrated that the Liver-Metastasis-Related Genes have high predictive potential for predicting the clinical outcomes of patients with pancreatic adenocarcinoma [17]. However, limited study on mRNA combination biomarkers for NSCLC metastasis has been done. We predicted in this study that differentially expressed genes linked to metastasis could be able to predict the prognosis of NSCLC patients. The mRNA expression data of NSCLC tissues in M0 stage and M1 stage from the TCGA datasets were combined in this study. Following cox and lasso regression, a six-gene prognostic signature was created with the potential to predict survival time for NSCLC patients.

## Materials and methods

### Data source

The transcriptome and clinical data were downloaded from the TCGA database (https://portal.gdc.cancer.gov/), including metastasis samples (n = 31) and non-metastatic samples (n = 733), and were used as training set. 117 LUAD samples from GEO datasets, the accession number of GEO datasets is GSE13213, and were used as external validation sets.

### Identification of DEGs

The ‘Limma’ package [18] in the R statistical software was used to identify DEGs between the metastatic group and the non-metastatic group, with adj *p* value < 0.05 set as the screening thresholds. A heat map cluster and volcano plot of the DEGs were created using the “pheatmap” and “ggplots” packages via R software.

### Gene ontology (GO) and Kyoto encyclopedia of genes and genomes (KEGG) analysis

To explore the potential functions of the metastasis-related gene signature, GO analysis and KEGG enrichment analysis were conducted by the ‘clusterProfiler’ [19] package. P.adjust < 0.05 were found to be statistically relevant.

### Univariate cox regression and lasso regression analysis

We first used the R package survival coxph function to perform Univariate Cox Regression analysis on DEGs to screen metastasis-related genes significantly related to the survival. *p* < 0.05 was selected as the threshold for filtering. Moreover, the screened prognosis-related metastasis-related genes were incorporated into Lasso regression model, in which penalties were applied to above gene for preventing overfitting effects of the model. We performed LASSO Cox Regression analysis and identified 12 signature genes [24]. At last, multivariate COX regression analysis constructed the prognostic model successfully. The patients of train and validation sets were divided into low- and high-risk groups on the foundation of the median value of the risk score of train cohort, respectively. Survival differences between the two groups were assessed by Kaplan–Meier. Meanwhile, univariate and multivariate prognostic analyses (*p* < 0.05) were performed for the training group to determine whether the riskScore obtained from the model could be an independent prognostic factor.

### Drawing and validation of the nomogram

A nomogram was established with the independent risk factors such as clinical information and risk score to predict the possibility of 1-year, 3-year and 5-year OS of NSCLC patients. The efficacy of the nomogram was evaluated by the calibration curve.

### Estimation of immune score, stromal score, and tumor purityimmune infiltration

The ESTIMATE package was used to calculate the immune score (representing the level of immune cell infiltration) and stromal score (representing the number of stroma) of each PAAD sample. The ESTIMATE score was defined as the sum of the immune and stromal scores. Then, the differences in stromal score, immune score, ESTIMATE scores, tumor purity scores between high-risk groups and low-risk groups were compared by the Wilcoxon test. *p* value < 0.05 was considered significant [16]. To predict the effect of immune checkpoint blockade therapy, we also explored the expression of immune checkpoint genes in the groups.

### Estimation of relationship between this prognostic risk model and clinical characteristics and tumor mutation burden (TMB)

We evaluated the relationship between the Risk score and clinical characteristics acquired from TCGA, as follows: M (M0 and M1), N (N0 and N1-3), T (T1-2 and T3-4), and stage (I-II and III-IV). The tumor mutational data of NSCLC patients were obtained from TCGA database, and tumor mutational burden (TMB) was calculated for each NSCLC patient.

### The analysis of GSEA

The R package “limma” was used to analyze differential expression between high-risk and low-risk groups [19], and all genes were ranked by fold change values. h.all.v7.4.symbols.gmt data set is downloaded from MSigDB, Gene Set Enrichment Analysis was performed to clarify the significant annotated pathways through R package “clusterProfiler".

## Results

### Identification of DEGs

**Fig. 1:**
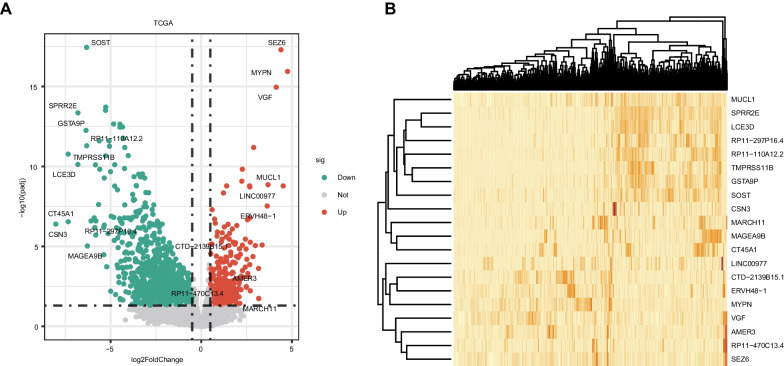
Identification of differential expressed gene between non-metastatic and metastatic group in NSCLC. **A** The volcano plot demonstrating the differentially expressed genes. **B** Heat map of differentially expressed genes in NSCLC

The 764 NSCLC samples in the TCGA dataset were separated into two groups: non-metastatic (31 samples) and metastatic (733 samples). The TCGA dataset yielded 2058 DEGs (Fig. 1A–B), 1499 of which were down-regulated and 559 of which were up-regulated.

### Functional enrichment analysis

**Fig. 2:**
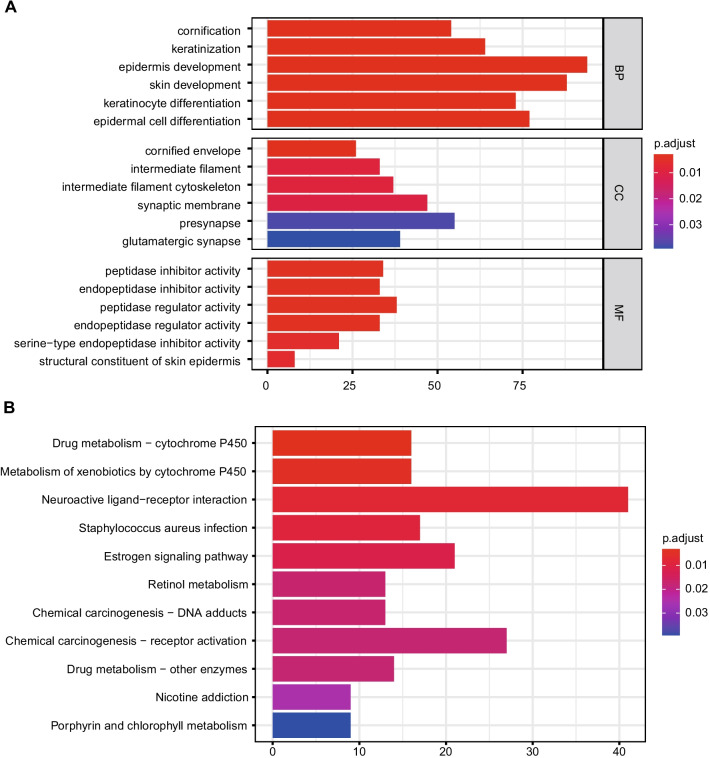
Representative results of GO and KEGG analyses. **A** The molecular functions of the 6 screened genes. **B** The potential biological pathways of the screened genes. Data from KEGG website (KEGG: Kyoto Encyclopedia of Genes and Genomes)

DEGs' biological functions and pathways can be studied via gene enrichment analysis. Epidermis development, skin development, epidermal cell differentiation, keratinocyte differentiation, and keratinization are among the biological processes enriched in GO (top 5). Presynapse, synaptic membrane, glutamatergic synapse, and intermediate filament intermediate filament are the biological components of GO (top 5). Peptidase regulator activity, endopeptidase regulator activity, endopeptidase inhibitor activity, peptidase inhibitor activity, and serine-type endopeptidase inhibitor activity are the top five molecular functions of GO (Fig. 2A). Similarly, neuroactive ligand-receptor interaction, chemical carcinogenesis-receptor activation, estrogen signaling route, staphylococcus aureus infection, and drug metabolism-cytochrome P450 are the top five significantly enriched pathways (Fig. 2B).

### Construction and validation of the risk score model based on 6 prognostic metastasis-related genes

**Fig. 3:**
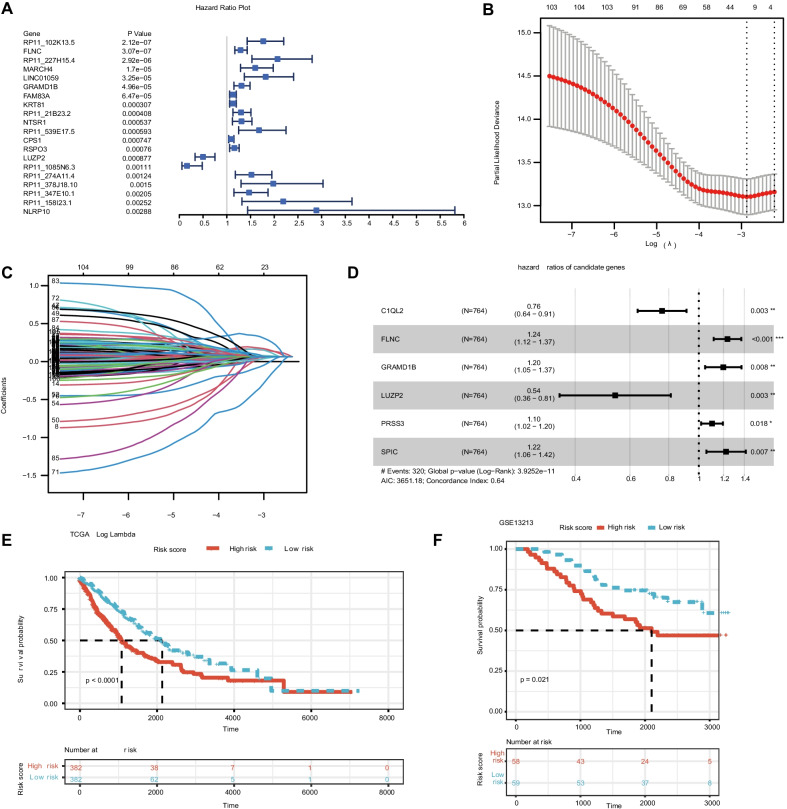
Construction of risk signature in the TCGA cohort. **A** Univariate Cox analysis of differentially expressed genes. (B) Cross-validation for tuning the parameter selection in the LASSO regression. **C** LASSO regression of the differentially expressed genes. **D** Multivariate Cox analysis of differentially expressed genes. **E**–**F** K–M survival analysis of risk prognostic model of NSCLC patients in TCGA

The DEGs in the TCGA training group were subjected to univariate Cox regression analysis. The findings of the univariate regression analysis revealed that genes related to metastasis were substantially correlated with NSCLC patients' prognosis (*p* 0.05). (Fig. 3A). For these genes having prognostic value, LASSO regression analysis was used to avoid over-fitting the prognostic model. The LASSO regression analysis revealed that 12 genes had a significant relationship with OS (Fig. 3B and C). Finally, we ran a multivariate regression analysis on the 12 genes we chose. C1QL2, FLNC, LUZP2, PRSS3, SPIC, and GRAMD1B were identified as risk variables for OS in the TCGA training group by multivariate regression analysis (Fig. 3D). The risk score was calculated as (− 0.265 × C1QL2) + (0.227 × FLNC) + (− 0.625 × LUZP2) + (0.095 × PRSS3) + (0.193 × SPIC) + (0.447 × GRAMD1B). Following that, the TCGA patients were split into high- and low-risk groups based on the median risk scores. Patients with high-risk scores had worse survival rates in the training set by the Kaplan–Meier curves (*p* 0.0001). (Fig. 3E). Similarly, 117 individuals from GSE13213 were chosen as the validation cohort and classified into high- and low-risk groups based on the median risk score, with the same risk score calculation formula as the TCGA cohort. The survival curve revealed a significant difference (*p* 0.05) between the two groups (Fig. 3F). The relationship between the RiskScore and clinical features was analyzed, and it was found that the risk score constructed based on the six-gene signature distinguished the high- and low-risk groups according to age, M0 stage, N stage, stage I-II, T1-2 stage. This finding consequently indicated that the risk model had a strong predictive ability across clinical features.

### The expression of RiskScore on different clinical features and the construction of nomogram

**Fig. 4:**
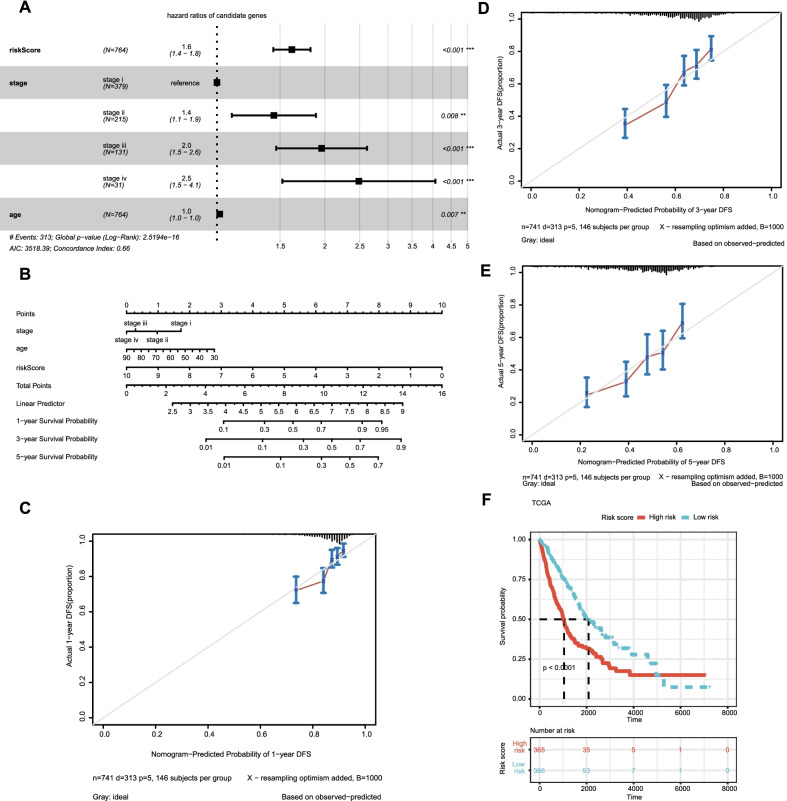
Construction and evaluation of prognostic models based on risk scores and clinical features. **A** Forest plot for multivariate COX regression analysis based on risk scores and clinical features. **B** A nomogram predicts the risk of progression in patients with NSCLC by four clinicopathological features. **C**–**E** The calibration curve is used to evaluate the accuracy of one-, three-, and five-year progress forecasts of nomograms. **F** K–M curves of prognostic models based on risk scores and clinical features

The multivariable Cox method was used to find the three independent prognostic indicators (age, stage, and risk score) of NSCLC patients in the TCGA data set (Fig. 4A). Following that, a nomogram for 1-year, 3-year, and 5-year survival rates was produced based on the age, stage, and risk score to objectively estimate the survival likelihood of each NSCLC patient (Fig. 4B). In addition, calibration curves for 1-year, 3-year, and 5-year survival rates were plotted to test the nomogram's accuracy, with the findings revealing that the nomogram-predicted and actual survival probability are generally in accord (Fig. 4C–E). Patients in the TCGA cohort were divided into high- and low-risk groups based on the median risk score computed from the nomogram. Figure 4F indicated that patients in the high-risk group had significantly shorter OS than those of the low-risk group (*p* < 0.001).

### The correlation between the prognostic risk model and clinical pathological characteristics of patients

**Fig. 5:**
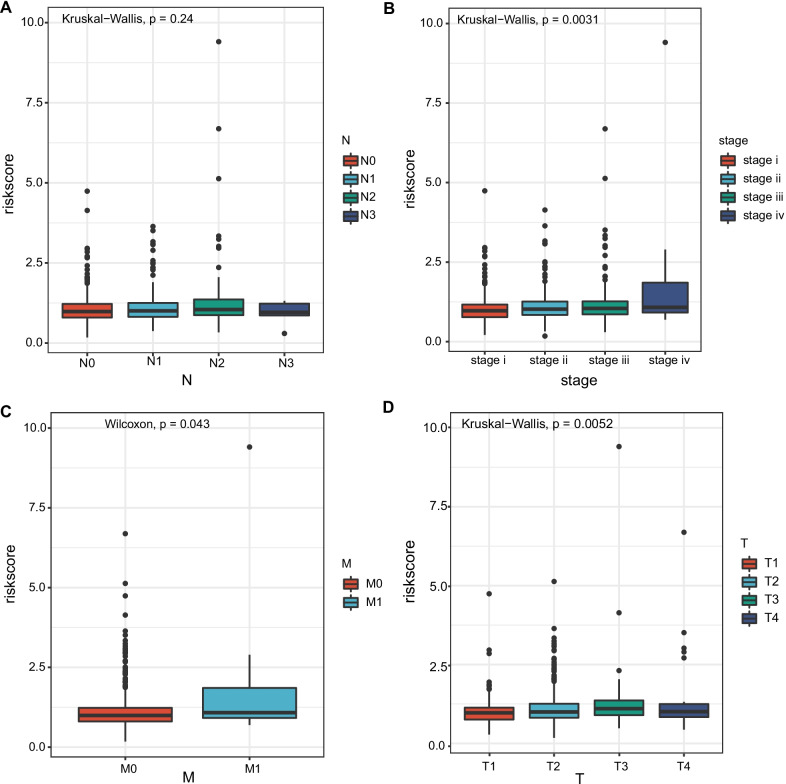
The correlation between the prognostic risk model and clinical pathological characteristics (stage, TNM) **A**–**D**

We started by looking at the association between risk scores and clinical variables. The results revealed that there was no significant difference in risk ratings among N stages (Fig. 5A). We looked at the differences in risk scores between different NSCLC groups. The subgroup analysis stratified by stage revealed that stage IV NSCLC patients had a significantly higher risk score than stage I NSCLC patients (*p* = 0.0031). (Fig. 5B). Furthermore, M1 NSCLC patients had a significantly higher risk score compared to M0 NSCLC patients (*p* = 0.043). In addition, T3NSCLC patients had a considerably higher risk score than T1 NSCLC patients (*p* = 0.0052). (Fig. 5C–D).

### Analysis of the relationship between the immune microenvironment and risk score model in NSCLC patients

**Fig. 6:**
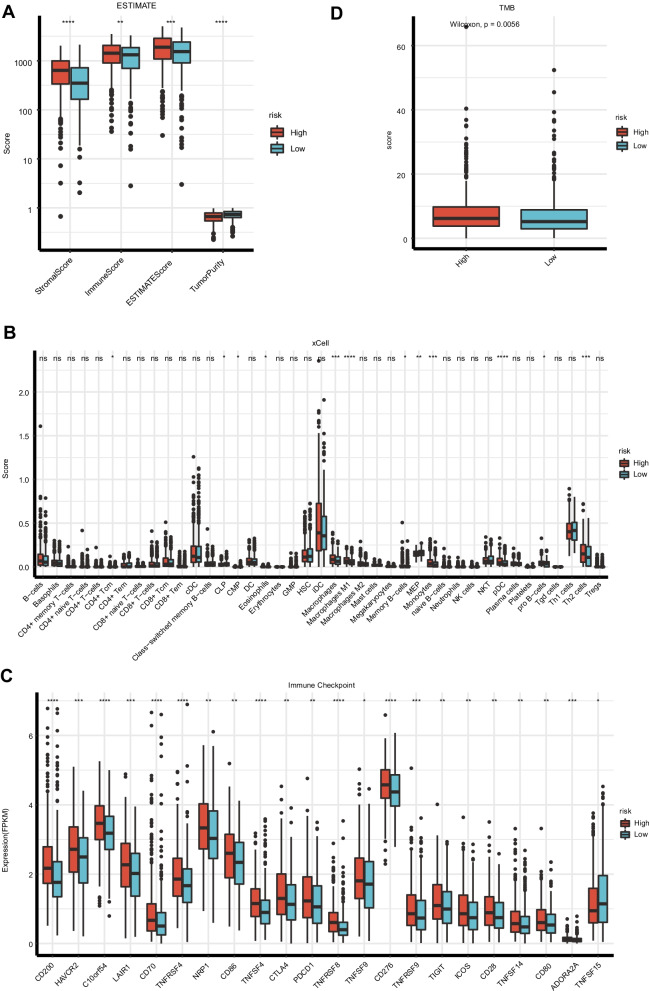
Analysis of the Relationship Between the Immune Microenvironment and Risk Score Model in NSCLC Patients. **A** ESTIMATE-analysis of the high and low risk groups. **B** Analysis of the immune-infiltrating cells. **C**Molecular analysis of immune checkpoints in high and low risk groups. **D** TMB scores for the high and low risk group

Using the ESTIMATE algorithm, we estimated the stromal score, immune score, and tumor purity of NSCLC from TCGA dataset. Our data showed that the immune score and stromal score of the high-risk group was significantly higher than those of the low-risk group (Fig. 6A), and the tumor purity score of the high-risk group was significantly lower than that of the low-risk group. To further explore the individual immune microenvironment and develop individualized treatment, immune infiltration and immune checkpoint genes in high- and low-risk group were further investigated (Fig. 6B–C). The low-risk group had considerably lower markers of Macrophages, Macrophages M1, MEP, Monocytes, pDC, and Th2 cells than the high-risk group. The low-risk group, on the other hand, showed increased Th1 cell, MEP, and HSC marker expression. In addition, variations in immune checkpoint genes were discovered in the high-risk and low-risk groups. TNFSF15 was expressed at higher levels in the low-risk group than in the high-risk group. When compared to the low-risk group, the high-risk group showed higher expressions of ADORA2A, TNFSF14, CD28, ICOS, TIGIF, TNFRSF9, CD276, TNFSF9, TNFRSF8, PDCD1, CTLA4, TNFSF4, CD86, NRP1, TNFRSF4, CD70, LAIR1, C10orf54, HAVCR2, and CD200.

We also estimated the TMB of each sample and discovered that in the TCGA dataset, TMB was significantly greater in the high-risk group (*p* = 0.0056). (Fig. 6D).

### GSEA analysis

**Fig. 7:**
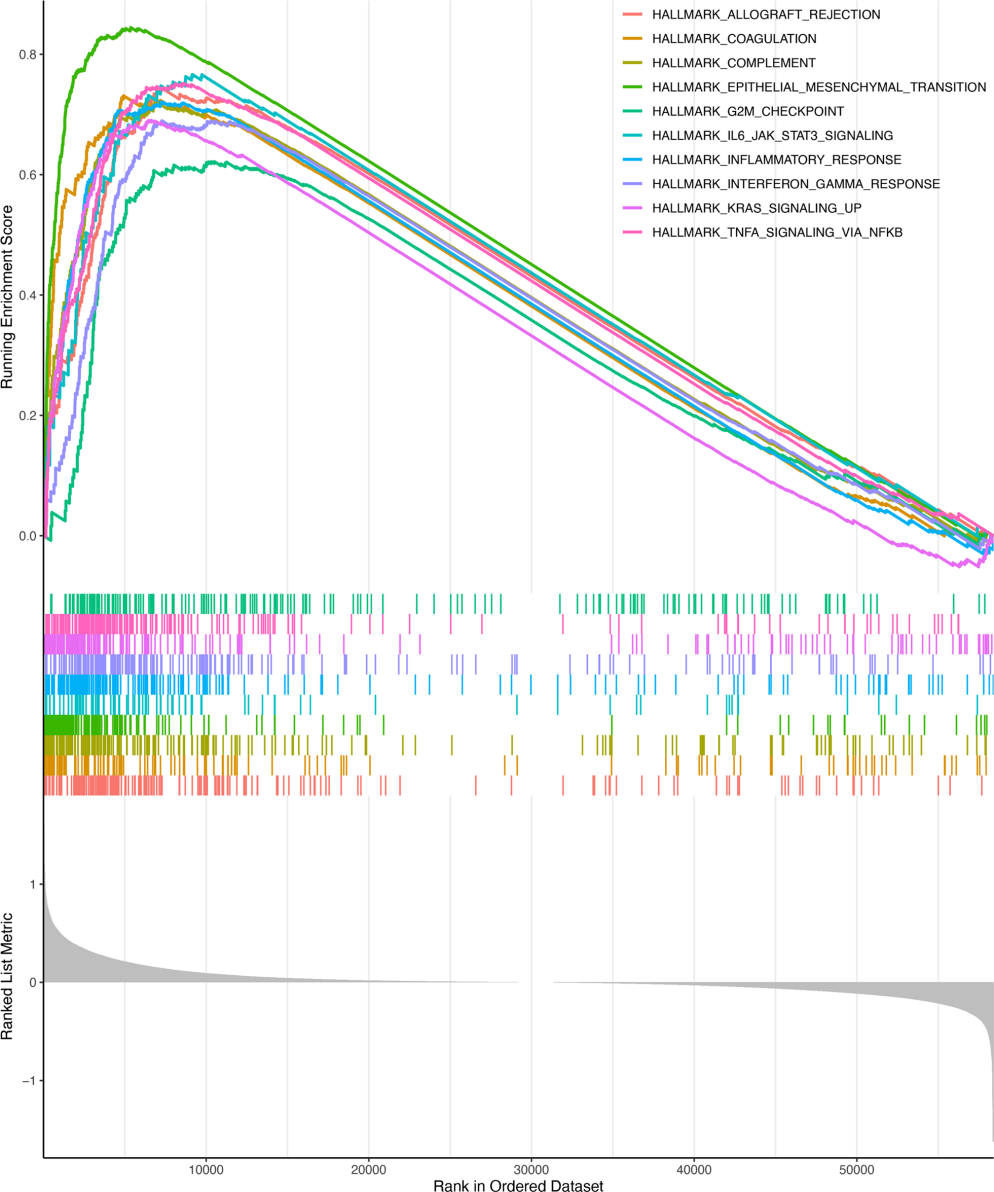
Gene Set Enrichment Analysis. Differences in gene sets between high and low risk groups

GSEA analyses were conducted to further explore the difference biological mechanism between low- and high-risk groups. we found that signaling pathway (Fig. 7), including allograft rejection, coagulation, complement, epithelial mesenchymal transition, G2M checkpoint, IL6-JAK-STAT3 signaling, inflammatory response, interferon gamma response, KRAS signaling up, TNFA signaling via NFkB were significantly enriched in the high-risk group.

## Discussion

In this article, NSCLC samples were classified into metastatic group and non-metastatic group according to M stage. TCGA was used as training cohort and construct a prognostic model, while GEO database was used as validation cohort to verify the efficacy of the prognostic model evaluation. Firstly, we analyzed the gene expression data and clinical data of NSCLC patients enrolled in TCGA, discerning 2058 DEGs related to metastasis. Using univariate, LASSO and multivariate Cox regression analysis, 6 mRNAs (*C1QL2, FLNC, LUZP2, PRSS3, SPIC, GRAMD1B*) had been found as independent prognosis predictors in NSCLC. Secondly, survival analysis was utilized to examine the availability of the prognostic model. The expression pattern of all the 6 mRNAs, had a correlation to OS which meant that with the generate of these mRNAs’ expression, patients would have a different survival time. Thirdly, the model constructed in training group was validated externally, adding dependability to the outcomes.

Through pathway enrichment analysis of metastasis-related genes, we found that many GO pathways were enriched, such as epidermis development, skin development, epidermal cell differentiation, keratinocyte differentiation, and so on. Many of them have been confirmed to be associated with tumor metastasis. Close relationship, for example, Sabounsji's study pointed out that the metastasis of NSCLC is closely related to epidermal cell differentiation [20]. A correlation between keratinocyte differentiation and Metastatic Melanoma was also pointed out in Li’s studies [21]. The mRNAs in model had been reported in other articles that they also had relationship with different types of cancers. A study from Sigin et al. found that in in luminal B breast cancer the methylation level of *C1QL2* is closely linked to neoadjuvant chemotherapy in luminal B breast cancer patients [22]. Filamin C (*FLNC*) is a large actin-cross-linking protein that is found in a variety of cells. According to the previous literature, temporary expression or silencing of *FLNC* can alter cancer cell proliferation and colony formation, whereas endogenous *FLNC* silencing can accelerate cancer cell motility and invasion [23]. *LUZP2*(leucine zipper protein 2 gene), located on Chr 11p13–11p14 and encoding a leucine zipper protein, has been shown to be deleted in Wilms' tumor patients. Wilms' tumor, genital abnormalities, aniridia, and mental retardation is a rare congenital abnormality syndrome characterized by Wilms' tumor, genital deformities, aniridia, and mental retardation [24, 25]. Furthermore, Zhao et colleagues found that *LUZP2* mRNA expression is elevated in hormone-naive prostate cancer (PC) relative to normal prostate tissues, but downregulated throughout the progression from hormone-naive PC to castration-resistant PC (CRPC) [26]. *PRSS3* (serine protease 3) is a member of the serine protease family that is produced in pancreatic acinar cells and released into the small intestine to help in digestion. According to Wang's findings, increased *PRSS3* expression may enhance stomach cancer metastasis and serve as an independent molecular indication of poor patient prognosis [27]. *SpiC* is a member of Spi subtypes, *SpiC* has crucial functions in myeloid differentiation, however, there have been no reports of the role of *SpiC* in tumors[28]. *GRAMD1B* (GRAM domain-containing protein 1B) was identified as a putative component of the signaling cascade17, has been implicated in human malignancies [29]. Specifically, it was reported to play a role in chemoresistance of ovarian cancer patients, such that *GRAMD1B* inhibition led to an anti-tumor effect [30]. Khanna’s study has proved that *GRAMD1B* regulates cell migration in breast cancer cells through JAK/STAT and Akt signalling [29]. Those results had represented similar conclusions as this study. 

Tumor metastasis is triggered by interactions between cancer cells and numerous stromal cell components of the tumor microenvironment, as well as by the accumulation of intrinsic changes in malignant cells [31, 32]. Inflammation and infiltration of tumor tissue by immune cells from the host, such as tumor-associated macrophages, myeloid-derived suppressor cells, and regulatory T cells, have been demonstrated to promote tumor development as well as invasion and metastasis [33, 34]. Our data showed that the immune score and stromal score of the high-risk group was significantly higher than that of the low-risk group. such as macrophages, macrophages M1, monocytes, pDC and Th2 cells immune infiltration was significantly higher than the low-risk group. This suggests that tumor metastasis-related genes also play a role in regulating tumor immunity. To explain more detailed immune cell infiltration in NSCLC, ssGSEA was used to find the low-risk group had higher marker expression of iDC, MSC, Th2 cells, Endothelial cells, Monocytes. These results are in line with the conclusions of previous studies [35, 36], indicating that our prognostic model can not only have a good predictive effect on the prognosis of patients with NSCLC. And it can respond to patient immune changes to some extent. This will be very important for immunotherapy with NSCLC patients. For example, in the future, patient response to immunotherapy can be predicted through prognostic models established in our study.

We wished to understand more genetically the possible mechanisms by which our model worked, GSEA was performed to do enrichment analysis of high and low risk groups separately, which could be found including allograft rejection, coagulation, complement, epithelial mesenchymal transition, G2M checkpoint, IL6 JAK STAT3 signaling, inflammatory response, interferon gamma response, KRAS signaling up, TNFA signaling via NFkB were significantly enriched in the high-risk group. These pathways have all been shown in previous studies to be directly or indirectly related to tumor metastasis. For example, EMT, an evolutionarily conserved developmental program, has been linked to carcinogenesis and imparts metastatic qualities to cancer cells by increasing mobility, invasion, and resistance to apoptotic stimuli. Furthermore, EMT-derived tumor cells have stem cell characteristics and are very resistant to treatment [37]. The cytokine interleukin-6 (IL6) and its downstream effector STAT3 form a major oncogenic pathway in breast cancer that has been hypothesized to be functionally linked to estrogen receptor (ER). Siersbak et al. found that IL6/STAT3 signaling promotes metastasis in ER + breast cancer that is not ER positive. A subset of ER enhancers is hijacked by STAT3 to produce a unique transcriptional pathway [38]. Some of the potential pathways we have identified have been reported to be associated with tumor metastasis, which validates our results, and our results find potential pathways that have not been explored to metastasis. This provides new perspectives for future studies of genes for tumor metastasis.

Finally, we developed a model and a biomarker for predicting the prognosis of NSCLC metastases by a series of bioinformation analyses. Patients in the low-risk category had a superior overall survival rate than those in the high-risk group, according to our findings which were confirmed in both the train and test cohorts. Our study opened a new avenue for the diagnostic and therapy of NSCLC. However, there were still exist some limitation in this research. Firstly, the data in TCGA may contain varying degrees of mistake, and the amount of data contained is limited, which may lead to inaccuracies. Second, the lack of in vivo and in vitro research will result in insufficient evidence. Last, There is still a flaw in our study that the TCGA database cannot provide paired samples. Therefore, we cannot longitudinally compare the situation of the same patient with different transfer times, and we will also include more cohorts in future studies to make up for this deficiency. It is also worth mentioning that our study is not based on all clinical features, including age, gender, etc., but a prognostic model constructed from only some accessible clinical features. Such as T and N staging and so on. Future studies need to incorporate more clinical features to achieve better model performance. As a result, further research and trials are needed to verify the model and biomarker to assure its robustness.

## Data Availability

The datasets generated and/or analysed during the current study are available in the TCGA repository. The Cancer Genome Atlas Program—National Cancer Institute. The validation set from GEO database, the accession number of GEO datasets is GSE13213. GEO Accession viewer (nih.gov).
